# Integrative analysis of DNA methylation suggests down-regulation of oncogenic pathways and reduced somatic mutation rates in survival outliers of glioblastoma

**DOI:** 10.1186/s40478-019-0744-0

**Published:** 2019-06-03

**Authors:** Taeyoung Hwang, Dimitrios Mathios, Kerrie L. McDonald, Irene Daris, Sung-Hye Park, Peter C. Burger, Sojin Kim, Yun-Sik Dho, Hruban Carolyn, Chetan Bettegowda, Joo Heon Shin, Michael Lim, Chul-Kee Park

**Affiliations:** 1 Lieber Institute for Brain Development, Baltimore, MD 21205 USA; 2 Department of Neurosurgery, Johns Hopkins University School of Medicine, Baltimore, MD 21287 USA; 3 Cure Brain Cancer Neuro-oncology Laboratory, Prince of Wales Clinical School, Lowy Cancer Research Centre, University of New South Wales, Sydney, Australia; 4 Department of Pathology, Seoul National University College of Medicine, Seoul, 03080 South Korea; 5 Department of Pathology, Johns Hopkins University School of Medicine, Baltimore, MD 21287 USA; 6 Department of Neurosurgery, Seoul National University College of Medicine, Seoul, 03080 South Korea; 7 Sidney Kimmel Comprehensive Cancer Center, Johns Hopkins University School of Medicine, Baltimore, MD 21287 USA; 8 BioFrontiers Institute, University of Colorado, Boulder, 80303 CO USA

**Keywords:** Glioblastoma, Long-term survivor, DNA methylation, Genome-wide analyses

## Abstract

The study of survival outliers of glioblastoma can provide important clues on gliomagenesis as well as on the ways to alter clinical course of this almost uniformly lethal cancer type. However, there has been little consensus on genetic and epigenetic signatures of the long-term survival outliers of glioblastoma. Although the two classical molecular markers of glioblastoma including *isocitrate dehydrogenase 1 or 2 (IDH1/2)* mutation and *O6-methylguanine DNA methyltransferase (MGMT)* promoter methylation are associated with overall survival rate of glioblastoma patients, they are not specific to the survival outliers. In this study, we compared the two groups of survival outliers of glioblastoma with *IDH* wild-type, consisting of the glioblastoma patients who lived longer than 3 years (*n* = 17) and the patients who lived less than 1 year (*n* = 12) in terms of genome-wide DNA methylation profile. Statistical analyses were performed to identify differentially methylated sites between the two groups. Functional implication of DNA methylation patterns specific to long-term survivors of glioblastoma were investigated by comprehensive enrichment analyses with genomic and epigenomic features. We found that the genome of long-term survivors of glioblastoma is differentially methylated relative to short-term survivor patients depending on CpG density: hypermethylation near CpG islands (CGIs) and hypomethylation far from CGIs. Interestingly, these two patterns are associated with distinct oncogenic aspects in gliomagenesis. In the long-term survival glioblastoma-specific sites distant from CGI, somatic mutations of glioblastoma are enriched with higher DNA methylation, suggesting that the hypomethylation in long-term survival glioblastoma can contribute to reduce the rate of somatic mutation. On the other hand, the hypermethylation near CGIs associates with transcriptional downregulation of genes involved in cancer progression pathways. Using independent cohorts of *IDH1/2*- wild type glioblastoma, we also showed that these two patterns of DNA methylation can be used as molecular markers of long-term survival glioblastoma. Our results provide extended understanding of DNA methylation, especially of DNA hypomethylation, in cancer genome and reveal clinical importance of DNA methylation pattern as prognostic markers of glioblastoma.

**Electronic supplementary material:**

The online version of this article (10.1186/s40478-019-0744-0) contains supplementary material, which is available to authorized users.

## Introduction

Despite advances in modern neuro-oncology, glioblastoma (GBM) continues to have a poor prognosis. Survival rates of adult GBM patients in the United States are quite low with 1-year, 2-year, 3-year, and 5-year relative survival rates estimated at 39.3, 16.9, 9.9, and 5.5%, respectively [26]. While the majority of GBM patients live no longer than 2 years, there is a subset of patients who live longer than 3 years and are classified as long-term survivors (LTS). This group of patients remains a puzzle to researchers in the field, as studies on clinical, radiological, histological, and molecular characteristics have yet to yield consensus regarding determinants of durable response to the current treatment [2, 3, 12, 14, 15, 20, 22, 24, 28, 29, 35–37]. For example, efforts to identify specific gene expression profiling patterns for LTS-GBM failed to uncover consistent features [8, 9, 30]. The classic genetic markers of favorable prognosis of GBM such as *O-6-methylguanine-DNA methyltransferase (MGMT)* promoter methylation or *isocitrate dehydrogenase (IDH)* mutation do not fully account for long term survivors of glioblastoma (LTS-GBM) [1, 9, 10, 21, 33, 39]. In particular, there are few studies for identification of molecular features associated with glioblastoma independent from *IDH* mutation or the *IDH* mutation-related signatures such as DNA methylation pattern called ‘Glioma CpG Island Hypermethylator Phenotype (G-CIMP)’ [8]. Although there is a report of concurrent gain of chromosomes 19 and 20 as a favorable prognostic factor for a subset of LTS-GBM that did not show G-CIMP, multiple other studies revealed no distinctive DNA copy number changes in LTS-GBM [9, 10, 30]. These results suggest that there is little chance to define LTS-GBM with a single genetic or epigenetic mechanism, emphasizing the importance of integrative understanding of molecular signatures in LTS-GBM. In fact, a recent integrated genomic analysis comparing LTS and short-term survivors (STS) GBM showed that multiple genetic and epigenetic factors are involved in divergent molecular features between the two extremes of the survival spectrum [28].

Although there have been some genome-wide studies for DNA methylation in survival outliers of brain cancer, most of them have largely focused on promoter regions and CpG islands (CGIs) in identifying aberrant methylation patterns or in classifying GBM due to its readiness of biological interpretation in terms of transcriptional regulation [16, 44]. However, the DNA methylation outside promotor-associated CGIs presents distinctive signatures in tumors and has significant effects on oncogenic pathways through multiple mechanisms. For example, DNA methylation of the CpG sites in gene body is known to be a major cause of cytosine to thymine transition mutations, as well as known to stimulate transcription elongation [11]. Moreover, there is a genome-wide crosstalk, not limited to genic region, between DNA methylation and histone modifications [19, 32]. One good example is that trimethylation of histone H3 lysine 9 (H3K9me3) is required for *DNMT3B* dependent de novo DNA methylation [32]. Therefore, unbiased analysis of DNA methylation across the whole genome is necessary to perform an integrative analysis of GBM of exceptional clinical course.

In the present study, we compared genome-wide DNA methylation profiles of *IDH* wild-type (IDH WT) GBM patients who lived longer than 3 years (*n* = 17, LTS-GBM) with the patients who lived less than 1 year (*n* = 12, STS-GBM). We found the striking differences in DNA methylation signatures between LTS- and STS- GBMs and performed integrative analyses for the differential patterns to understand their functional implications on epigenetic aspects of GBM. Also, we evaluated the classification potential of our identified patterns of DNA methylation with independent cohorts of mostly IDH WT GBM patients to test their possibility as prognostic marker.

## Materials and methods

### Patient samples

Survival data of patients with histologically-confirmed GBM at Seoul National University Hospital, Korea between 2000 and 2010 were obtained by retrospective review of patients’ charts, and from National Cancer Registry survival database of Korea. We identified 34 out of 429 newly diagnosed patients who lived longer than 3 years, and 17 patients were finally selected for discovery cohort of LTS-GBM group after central histological review. The central histological review included a complete agreement of histological diagnosis between two neuropathologists (S.H.P. and P.B.) on their independent slide review, and no evidence of *IDH1* mutation on immuno-histochemical evaluation. For the comparison, 12 GBM patients who lived less than 1 year in spite of standard care were chosen for the STS-GBM group. For the validation cohort, 10 GBM samples were obtained from patients treated at Prince of Wales Clinical School, Australia between 2004 and 2009 who lived longer than 3 years. This cohort contained 5 wildtype *IDH1*, 1 mutated *IDH1*, and 4 patients with unknown *IDH1* status. Patients’ clinical characteristics are summarized in the Additional file 1: Table S1. We also collected IDH WT samples that provide raw data of Illumina’s Infinium Human Methylation450K BeadChips from the cancer genome atlas (TCGA) dataset: 3 LTS and 39 STS samples.

### DNA extraction

DNA was extracted by standard methods for formalin-fixed paraffin-embedded tumor tissue. The QIAamp DNA FFPE Tissue Kit (Qiagen) was used to isolate and purify DNA from formalin fixed, paraffin embedded tissue. The protocol suggested by the manufacturer was used to isolate DNA. The quality and quantity of DNA was assessed both by Nanodrop and Bioanalyzer technology. The isolation of DNA from all clinical samples was performed by the Genetics Core Store of Johns Hopkins School of Medicine.

### Genome-wide DNA methylation measurement

DNA methylation was measured by using Illumina’s Infinium Human Methylation450K BeadChips (“450K bead array”) according to the manufacturer’s manual. DNA bisulfite conversion was carried out using EZ DNA Methylation Kit (Zymo Research) by following manufacturer’s manual with modifications for Illumina Infinium Methylation Assay. Briefly, 400 ng of genomic DNA was first mixed with 5 ul of M-Dilution Buffer and incubated at 37 °C for 15 min and then mixed with 100 ul of CT Conversion Reagent prepared as instructed in the kit’s manual. Mixtures were incubated in a thermocycler with 16 thermal cycles at 95 °C for 30 s and 50C for 1 hour. Bisulfite-converted DNA samples were loaded onto 96-column plates provided in the kit for desulphonation and purification. Concentration of eluted DNA was measured using Nanodrop-1000 spectrometer.

### Preprocessing of DNA methylation array data: filtering probes and intra-sample normalization

We filtered the following probes in the 450K bead array: i) probes whose detection *p*-values are greater than 0.01 in more than 5% of samples. According to Illumina, the detection *p*-value of a probe is calculated by comparing the intensity of target probes (the sum of methylated bead intensity and unmethylated bead intensity) with the intensities of negative control probes, ii) the probes whose bead counts are less than 3 in more than 5% of samples, iii) probes of non-CpG sites, iv) probes with SNP sites, v) probes whose sequences align to multiple locations in the reference genome, vi) probes associated with the sex chromosomes. The list of SNP-spanning probes and multiple-aligned probes were obtained from the R package “ChAMP” [23]. The 407,351 probes among the total of 485,512 probes in the array were passed in the above filtering. The methylation level of a probe is represented by the “beta” value that is defined as a ratio of the intensity of methylation bead to the sum of total intensity of probe and the offset value of 100.

In the 450K bead array, a probe measures DNA methylation level with either ‘type I’ assay or ‘type II’ assay. While the probe with type I assay employs two different bead types for methylation and unmethylation with the same color channel, the probe with type II assay uses two different colors with one bead type to measure methylation and unmethylation status. Since the two types of probes measure DNA methylation with different chemistries, it is necessary to adjust technical variation between them. We adopted a beta-mixture quantile dilation (BMIQ) method proposed by Teschendorff et al. [40], to perform normalization of beta values within a sample.

### Computational analyses

Differentially methylated sites were identified between LTS- and STS- GBM groups by using R package, RUV-inverse for DNA methylation [18]. Briefly, this package evaluates beta values for a probe with generalized least squares regression after controlling batch effects based on negative control probes in the array. The sites were marked as significant differential methylation if their corrected *p*-values for multiple testing by false discovery rate (FDR) were less than 0.01.

Pearson correlation coefficient was calculated between DNA methylation level (beta) for a given site and RNA expression level (log2 transformation of FPKM+ 1) of the corresponding refSeq mRNA using 32 GBM samples that are available for both of RNA-seq and 450K bead array in TCGA. When multiple sites were matched to a single gene, the maximum value of correlation coefficient was assigned for a gene. We only considered the sites whose standard deviation of beta values is greater than 0.1 and mean value of FPKM is greater than 0 to focus on the sites with enough variation of DNA methylation and detectable gene expression across the GBM samples.

The list of somatic mutation in GBM was downloaded from TCGA for 136 samples that have both of whole exome sequencing and 450K bead array. In each sample, a somatic mutation is associated with a probe site if the mutation is found between 5 K base-pairs upstream and downstream of a probe site.

Gene ontology (GO) analysis for a given set of sites on 450K bead array was performed adjusting for the selection bias of genes inherent from non-uniform distribution of probes across genome. We took a weighted resampling approach similar with the previous report [43]. Specifically, 1000 random sets of as many genes as the test set were generated with selection of a gene weighted according to the number of probes assigned to a gene. Statistical significance of a GO term was determined by comparing the number of genes associated with the GO term between the test set and the randomly-selected gene sets. Significant GO terms were selected among the category of biological process based on FDR threshold of 0.05.

### Probe annotation and open source datasets

The probes on the 450K bead array were annotated with R package, “IlluminaHumanMethylation450kanno.ilmn12.hg19”, except for their assignment to genes that were determined by the software ANNOVAR [42] using NCBI reference genes (Refseq). Note that the probes were grouped into four categories according to the distance from the closest CGI in the UCSC annotation (Additional file 1: Figure S1): i) “island” if a probe is inside CGI, ii) “shore” if a probe is within 2000 bases, iii) “shelf” if a probe is between 2000 and 4000 bases, iv) “open sea” otherwise. Further categories were made by distinguishing 5′ and 3′ direction from the closest CGI denoted by ‘N_’ and ‘S_’ respectively such as ‘N_shore’ and ‘S_shore’. The probes were also grouped according to the distance from transcription start sites (TSS) of the NCBI reference genes: i) “TSS1500” if a probe is within 200–1500 bases upstream TSS, ii) “TSS200” if a probe is between 0 and 200 bases upstream TSS, iii) “1st exon” if a probe is associated with first exon, iv) “Far” otherwise. If a probe has multiple categories, the following priority is applied to determine its single category: 1st Exon>TSS200 > TSS1500 > Far.

We obtained epigenetic annotation of human genomes from the ENCODE project [7, 38], specifically the regions of ChIP enrichment for 12 histone modifications (H2az, H3K27ac, H3K27me3, H3K36me3, H3K4me1, H3K4me2, H3K4me3, H3K79me2, H3K9ac, H3K9me1, H3K9me3, H4K20me1) across 66 cells (Additional file 1: Figure S2). The results from uniform processing by ENCODE Analysis Working Group were downloaded and manually parsed for further analyses. Also, we obtained H3K9me3 ChIP-seq and 450K bead array datasets of H1 from the ENCODE project.

TCGA datasets were downloaded for the level 3 data of RNA sequencing (RNA-seq), Illumina Infinium HumanMethylation450 BeadChip and exome sequencing of available GBM samples: FPKM, beta values and the compiled list of somatic mutation sites respectively.

For U87MG, a GBM cell line, DNA methylation profiled by 450K bead array was obtained from ENCODE project. ChIP-seq peak (q-value< 0.05) of H3K27ac was provided from [13] (GSE36354).

## Results

### Differential DNA methylation patterns between LTS- and STS-GBM

**Fig. 1:**
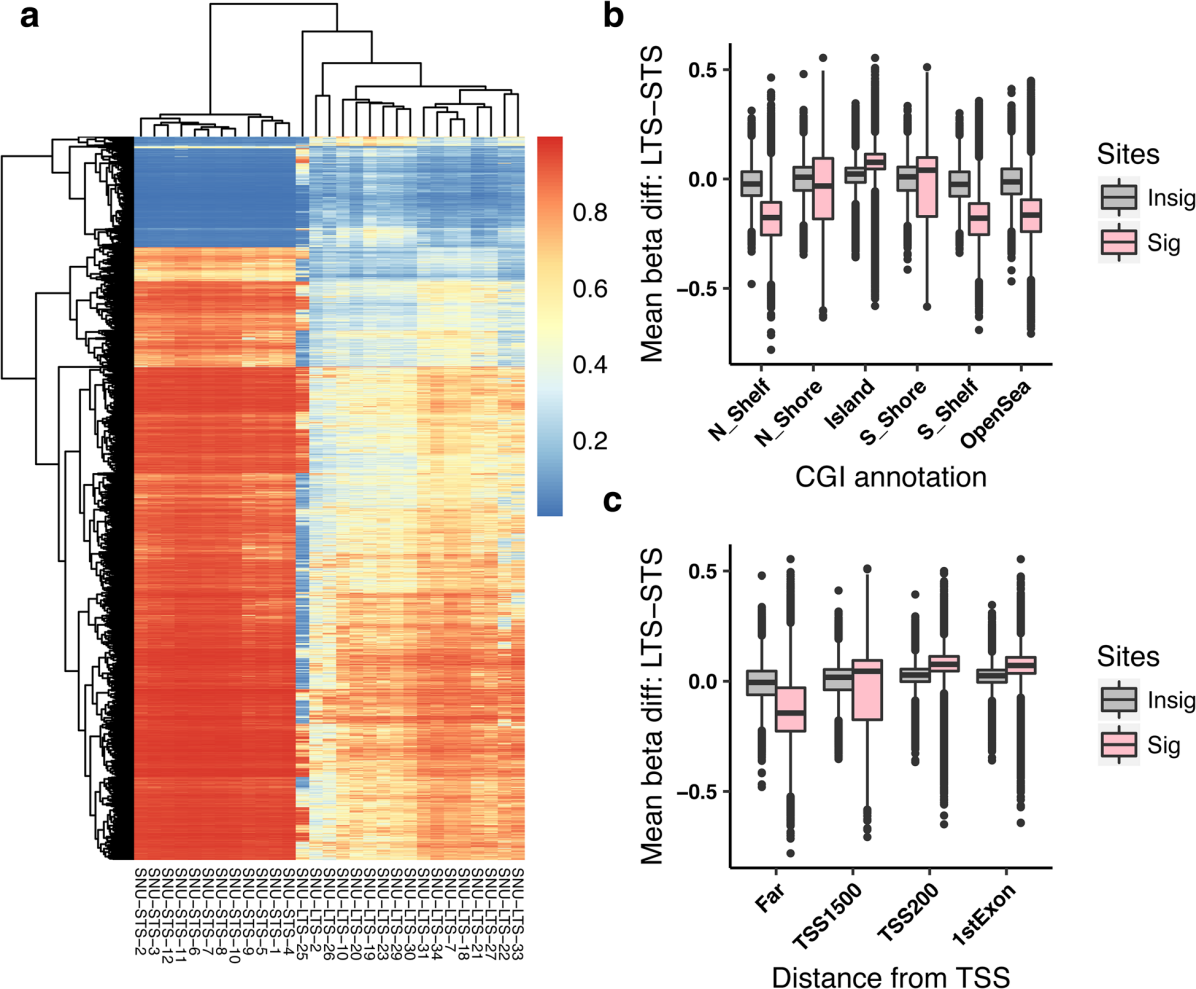
Differentially methylated sites between LTS-GBM and STS-GBM. **a** Heatmap of DNA methylation levels measured as beta values: color gradients from blue to red correspond to beta values from 0 to 1. Hierarchical clustering was performed for both of glioblastoma patients (columns) and the differentially-methylated sites between LTS-GBM and STS-GBM (rows). Here, hierarchical clustering of the most significant sites (−log_10_(FDR) < 5: 13,049 sites) with Euclidean distance and complete linkage was performed for convenient visualization. **b** Distribution of the mean increased level of DNA methylation in LTS relative to STS (labelled as “LTS-STS” in y-axis) across the sites categorized by their locations from the closest CpG Island (CGI) (see materials and methods). The group of selected significant sites are denoted by pink while the other sites are described by grey. If the distribution is shifted to the positive in y-axis (for example, at “Island”), it means that LTS has in general higher DNA methylation compared to STS while the shift to the negative in y-axis indicates the opposite case (for example, in “OpenSea”). **c** Same as (**b**) except that categorization of sites were done by distances from the nearest transcription start site (TSS) (see materials and methods)

The statistical tests comparing the methylation status of 17 IDH WT LTS-GBM with those of 12 IDH WT STS-GBM identified 161,794 autosomal sites showing the significant differences (see material and methods, FDR < 0.01) in methylation levels (Fig. 1a and Additional file 2: Table S2). They generally show moderate differences in methylation level such that most of the significant sites have mean differences of beta values less than 0.3 (Additional file 1: Figure S3). Differentially methylated sites are evenly distributed throughout the chromosomes (Additional file 1: Figure S4). Interestingly, the significant sites tend to have higher methylation level in LTS when they are closer to CGI or transcription start site (TSS), while lower methylation was observed in the distant region from island or TSS in LTS compared with STS (Fig. 1b and c).

**Fig. 2:**
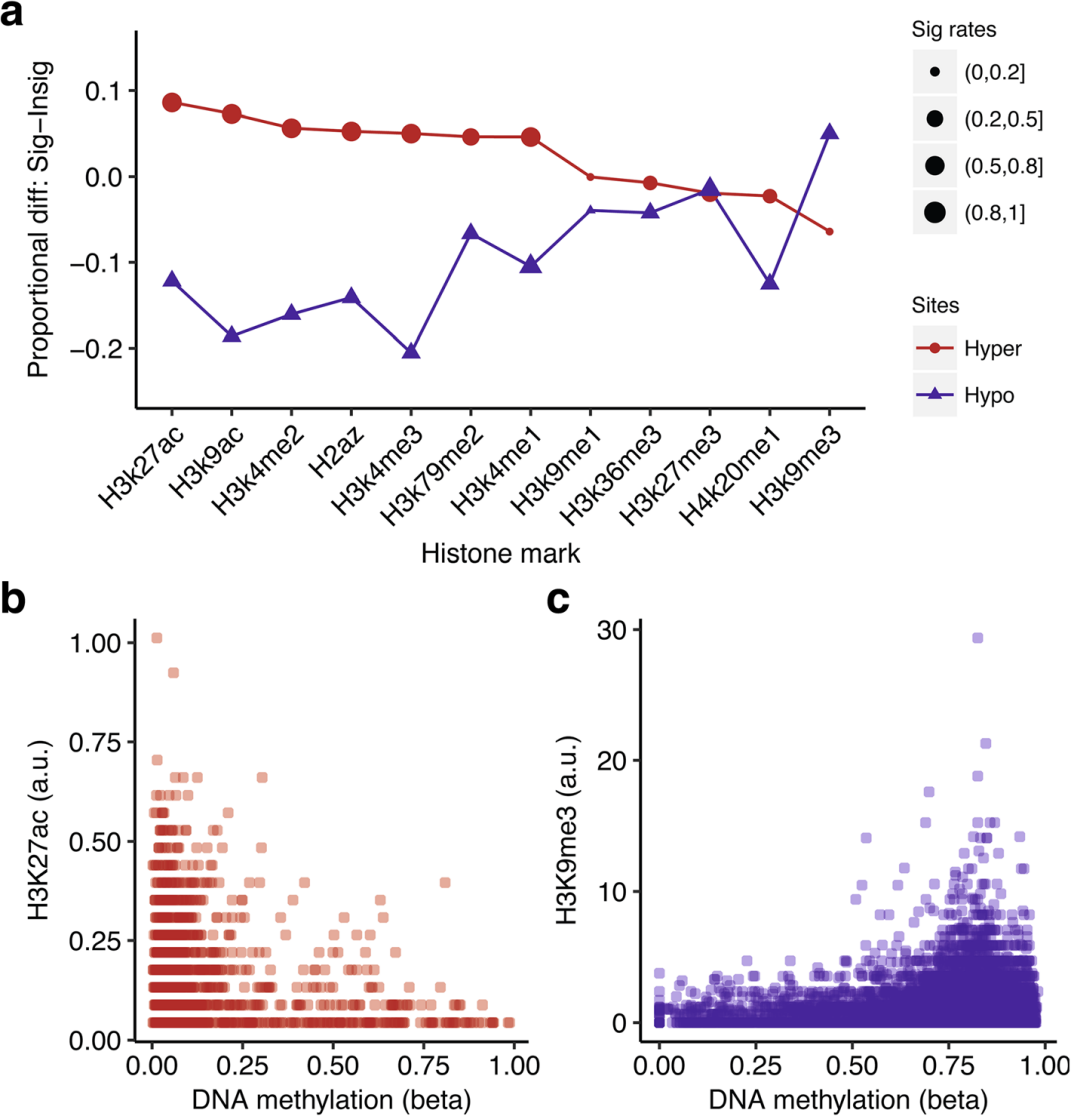
Enrichment of differentially-methylated sites in regulatory histone marks. **a** Enrichment patterns of differentially-methylated sites with histone marks. For a given histone mark (x axis), Y axis value describes the differences of enrichment proportion between the significantly- differential methylation sites and the insignificant sites. The red denotes hypermethylated sites in island while the blue describes hypomethylated sites in open sea. The size of dot indicates the range of enrichment proportion of the significantly- differential sites. We grouped the proportion to 4 regions for clear visualization (4 dot sizes): 0~0.25, 0.25~0.5, 0.5~0.75, 0.75~1. **b** The relation between the ChIP-seq signal of H3K27ac and the DNA methylation level (beta) for the hyper-methylated sites in U87MG cell line. Each dot denotes the site. **c** The same plot as (**b**) for the hyper-methylated sites in H1 cell line

In order to understand the differential patterns in greater detail, we investigated the association of the identified patterns with histone modifications. We focused on the representative sites of the two patterns of significant sites: the sites inside CGI (‘Island’) that are hypermethylated and the sites that are hypomethylated in the ‘open sea’ that are far from CGI. Here, hypermethylation or hypomethylation indicated higher or lower DNA methylation levels in LTS relative to STS. The two groups of representative sites were constructed by selecting the sites whose mean difference of beta values between LTS-GBM and STS-GBM was greater than 0.2 in each region, then were correlated with the genomic regions of histone marks in the ENCODE consortium dataset (see materials and methods). The two groups showed distinct enrichment profiles of histone marks relative to the insignificant methylation sites (Fig. 2a). For example, H3K27ac was most enriched with hypermethylated sites in island, however, this was underrepresented in the hypomethylated sites in the open sea. On the contrary, H3K9me3 was mostly enriched in the significant sites in the open sea while it was depleted in the island. Furthermore, using the publicly- available datasets (ENCODE, TCGA and Lin et al. 2012) regarding a glioblastoma cell line, U87MG, we found that the DNA methylation levels were negatively correlated with the H3K27ac levels at the hypermethylated sites in island (Fig. 2b, coefficient of linear regression: − 0.16 with *N* = 1298, *p*-value< 10^− 15^). Although the DNA methylation levels at the hypomethylated sites did not correlate with H3K9me3 levels in U87MG (Additional file 1: Figure S5), we confirmed the positive correlation between DNA methylation levels and H3K9me3 levels at the hypomethylated sites in a ENCODE H1 cell line (Fig. 2c, coefficient of linear regression: 0.29 with *N* = 24,372, *p*-value< 10^− 15^). These results imply that our comparison between LTS-GBM and STS-GBM identifies differential DNA methylation signatures with regulatory potential related to specific histone marks. In fact, both histone marks of H3K27ac and H3K9me3 are known to be related with DNA methylation [5].

### Functional implications of LTS-GBM-specific DNA methylation patterns

**Fig. 3:**
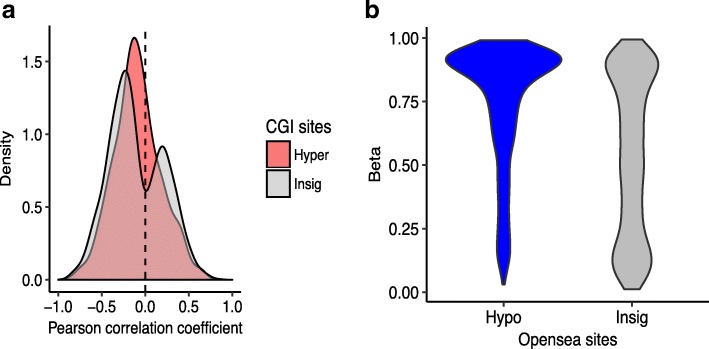
Functional implication of hyper- and hypo-methylated sites in LTS-GBM. **a** Distribution of Pearson correlation coefficients between DNA methylation level (beta) and gene expression measured by RNA-seq (FPKM) across 32 GBM samples in TCGA. The red describes the genes (number of genes: 366) corresponding to the selected hyper-methylated sites in this study while the gray shows the genes (number of genes: 4413) matched to insignificant sites associated with the island. **b** DNA methylation level around somatic mutations found in 136 TCGA GBM samples by whole exome sequencing. The hypo-methylated sites in LTS-GBM (*N* = 1802) were compared with the insignificant sites in open sea (*N* = 5032)

Enrichment of histone marks of active transcription such as H3K27ac and H3K9ac in hypermethylated sites inside CGI suggests their potential in transcriptional regulation. These sites are also enriched in promoter regions (Additional file 1: Figure S6a). In fact, it is well known that promoter region is represented by histone marks of H3K9ac and H3K27ac. As the role of DNA methylation in the promoter region is known to repress transcription, we tested whether DNA methylation of these sites is associated with varying gene expression levels. Specifically, the distribution of correlations between DNA methylation and gene expression across GBM samples in the TCGA were compared between these sites and the insignificant genic sites in the CGI. The distribution of Pearson correlation coefficients was shifted to the left to the 0 value in the hypermethylated sites while the non-significant sites in CGI showed bimodal distribution (Fig. 3a), indicating the enrichment of negative correlation between DNA methylation and gene expression for the sites showing the hypermethylated sites. We also performed gene ontology (GO) analyses to understand genes regulated by hypermethylation in LTS-GBM. Genes associated with hypermethylated sites in CGI were enriched with the gene ontology terms related to cancer progression such as cellular proliferation and cellular attachment (Table 1). These results collectively imply that hypermethylation around CGI found in LTS-GBM suppress gene expression in tumor progression pathways.

**Table 1:** Gene Ontology (GO) results of the hyper-methylated sites

ID	Description	Number of genes (intersection/ total)	Significance (*p*-value, FDR)
GO:0008283	The multiplication or reproduction of cells, resulting in the expansion of a cell population.	50 / 263	0, 0
GO:0016338	The attachment of one cell to another cell via adhesion molecules that do not require the presence of calcium for the interaction.	6 / 13	0, 0
GO:0071364	Any process that results in a change in state or activity of a cell (in terms of movement, secretion, enzyme production, gene expression, etc.) as a result of an epidermal growth factor stimulus.	12 / 33	0.004, 0.019

ID: GO ID, Description: GO Term description, Number of genes: “total” is the number of genes that are mapped in the 450 K bead array in the GO term, “intersection” means the number of genes that have associations with the selected hyper-methylated sites among the total, Significance: “*p*-value” was obtained from permutation test, “FDR” is False Discovery Rate (see materials and methods)

The hypomethylated sites in the open sea were slightly enriched with gene body region (Additional file 1: Figure S6b). Also, the they showed the enriched positive correlations between DNA methylation and gene expression, being consistent with the well-known association of DNA methylation with gene expression in gene body [11] (Additional file 1: Figure S7). However, their effect was not obvious as much as the hypermethylated sites. In addition, the hypomethylation-associated genes were not enriched with GO terms related to cancer (Additional file 1: Table S3). We concluded that hypomethylation in LTS-GBM is not involved with gene activity of oncogenic pathway, at least as much as hypermethylation.

Previous studies suggest that epigenetic modifications of the genome can affect the mutational rate in the human genome [17]. We hypothesized that hypomethylation in the open sea might be related with local mutational rate. Using the exome sequencing data of GBM in TCGA, we looked at DNA methylation levels around the somatic mutation in the open sea (see materials and methods). Interestingly, we found that the distributions of DNA methylation levels around somatic mutations are different according to the sites in the open sea (Fig. 3b). In the LTS-GBM specific sites, somatic mutations were preferentially observed when they were highly methylated while the other sites in the open sea showed little bias of DNA methylation. We also looked into the relative location of the sample with somatic mutation in the distribution of DNA methylation levels (136 samples) by calculating Z-score of DNA methylation level. The hypo-methylated sites tend to have higher DNA methylation levels in the sample with somatic mutation relative to the mean DNA methylation levels across the 136 samples that were interrogated here (Additional file 1: Figure S8). This tendency is weaker in the insignificant sites (Kolmogorov-Smirnov test, number of hypo-methylated sites:1802, number of insignificant sites: 5032, *p*-value < 2.2e-16). These results imply that the hypomethylation in LTS-GBM occurs at the sites potentially contributing to lower somatic mutation rates.

### Validation of differential methylation in an independent LTS-GBM cohort

**Fig. 4:**
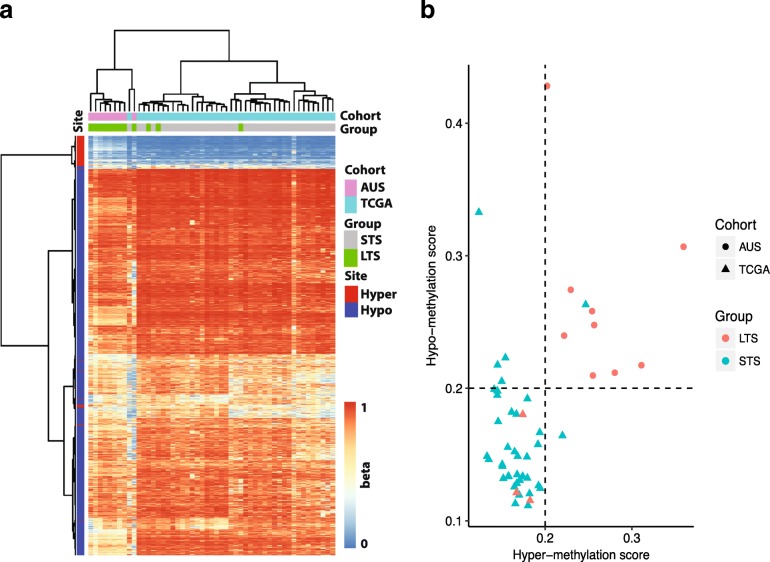
Hyper- and hypo-methylation in two independent cohorts (TCGA and ‘Australian’). **a** Heatmap of DNA methylation levels measured as beta values: hierarchical clustering was performed for both of samples (columns) and the sites (rows) either hyper- or hypo-methylation identified in the discovery cohort (“SNU”). The color gradients from blue to red correspond to beta values from 0 to 1. **b** Summary scores in terms of hyper- and hypo-methylation for each sample in two test cohorts: Each sample, denoted by a dot is assigned to two simple arithmetic averages (x and y axes values) of beta values in hyper- and hypo- methylation sites. The dashed line indicates 0.2 as a decision threshold for LTS-GBM

Finally, we asked if our identification of hypermethylation in CGIs and hypomethylation in open sea is reproducible in independent cohorts of LTS-GBM. Hierarchical clustering was performed for two independent test sets of IDH WT GBM samples (TCGA and ‘Australian’, see materials and methods) using DNA methylation level at the identified hyper or hypomethylated sites (Fig. 4a). First, all of 9 Australian LTS samples show similar pattern of hyper and hypomethylation. Second, 38 out of 39 STS in the TCGA replicated our pattern while all 3 LTS-GBM samples were not accounted for by our pattern.

We also attempted to assign scores to each test sample in terms of hypermethylation and hypomethylation in order to predict LTS-GBM. The simple arithmetic average of beta values in each pattern is assigned to a sample. We predicted a sample as LTS-GBM when its mean beta values for both of hypermethylation and hypomethylation are greater than 0.2, otherwise it was called as STS-GBM. As a result, 9 out of 12 LTS-GBM were predicted correctly (sensitivity: 75%) and 38 out of 39 STS samples are correctly recalled (specificity: 97%) (Fig. 4b).

## Discussion

Epigenetic aberrations are increasingly regarded as a gateway to neoplastic transformation in gliomas [16]. In particular, a recent study showed that DNA methylation was the strongest predictor of prolonged survival in GBM compared to any of clinical variables, RNA expressions for mRNA/miRNA, and the available genomic data including germline/somatic point mutation and copy number variation [14]. They found the importance of DNA methylation based on the statistical analyses for clinical data and multimodal molecular profiles of 44 patients (7.4%) who lived longer than 3 years among 591 GBM patients from TCGA dataset. However, the effect of DNA methylation is often confounded with genetic perturbation. For example, although G-CIMP signatures were found to be a favorable prognostic marker of GBM [4, 25], the majority of them overlap with *IDH* mutation [27, 41]. Therefore, it is important to evaluate the effect of DNA methylation in LTS-GBM after controlling genetic background such as *IDH* mutation. There have been several studies identifying DNA methylation signatures specific for IDH WT LTS-GBMs. Mock et al. compared global DNA methylation profiling using Methyl-CpG-Immunoprecipitation in 14 LTS and 15 STS-GBM patient samples with *IDH1* wild-type, and found that hypermethylation of multiple CpGs mapping to the promoter region of *LOC283731* correlated with improved patient outcome [22]. Zhang et al. analyzed methylation profiles of 13 LTS and 20 STS-GBM patients using Illumina Infinium Human Methylation 27 K Bead-Chips [44]. They identified the promoter methylation in *ALDH1A3* is a prognostic biomarker in a *IDH1* wild-type and unmethylated *MGMT* promoter GBM sample. However, these studies only focused on DNA methylation in promoter regions and did not provide comprehensive understanding of landscape of DNA methylation signatures in LTS-GBM.

In the present study, we pursued the genome-wide understanding of DNA methylation patterns specific to IDH WT LTS-GBM. We showed that LTS-GBM, compared with STS-GBM, is characterized by hypermethylation in the CGI and hypomethylation at open sea throughout the genome. It is well known that methylation of CGIs at promoter region is linked to silence of gene expression, and our results were consistent with this idea and previous findings of hypermethylation in LTS-GBM. However, hypomethylation of open sea in LTS-GBM is poorly appreciated so far. It is not compelling to understand this pattern in terms of gene activity since our GO analysis showed that the genes are not generally related with cancer. Also, as genome-wide hypomethylation in cancer is known to be ubiquitous feature of carcinogenesis [6], the hypomethylation pattern of LTS-GBM relative to STS-GBM seems to be enigmatic. However, a previous study showed that methylated cytosine bases are prone to mutation by spontaneous deamination to thymine [31]. Additionally, there is evidence linking levels of regional mutation density of cancer cells with the heterochromatin-associated H3K9me3 that is a sole histone mark enriched with our hypomethylated sites [34]. Our analysis of exome-sequencing of GBM showed that local somatic mutations tend to occur near the LTS-GBM-specific sites in open sea when these sites have higher DNA methylation levels. This suggests that significantly lower methylation around the identified sites in LTS-GBM can reduce the risk of de novo mutation contributable to oncogenesis possibly in the absence of proper DNA repair mechanisms in GBM. However, the mechanism of how the hypomethylation in the region distant from CGI in LTS-GBM affects the rates of somatic mutation and contributes to better survival relative to STS-GBM remains to be determined by further studies.

We also tested whether the two identified genome-wide patterns were able to serve as molecular markers of LTS-GBM by applying the identified sites to the independent cohorts of LTS samples. Although we confirmed the reasonable performance of the sites with the simple rule of prediction, the LTS samples from TCGA cohort did not show the predicted pattern. This might be due to small sample size (*N* = 3) or unknown batch effect. We anticipate a  larger cohort as well as better prediction rules in the future study to validate applicability of the identified methylation patterns specific to LTS-GBM.

## Conclusion

**Fig. 5:**
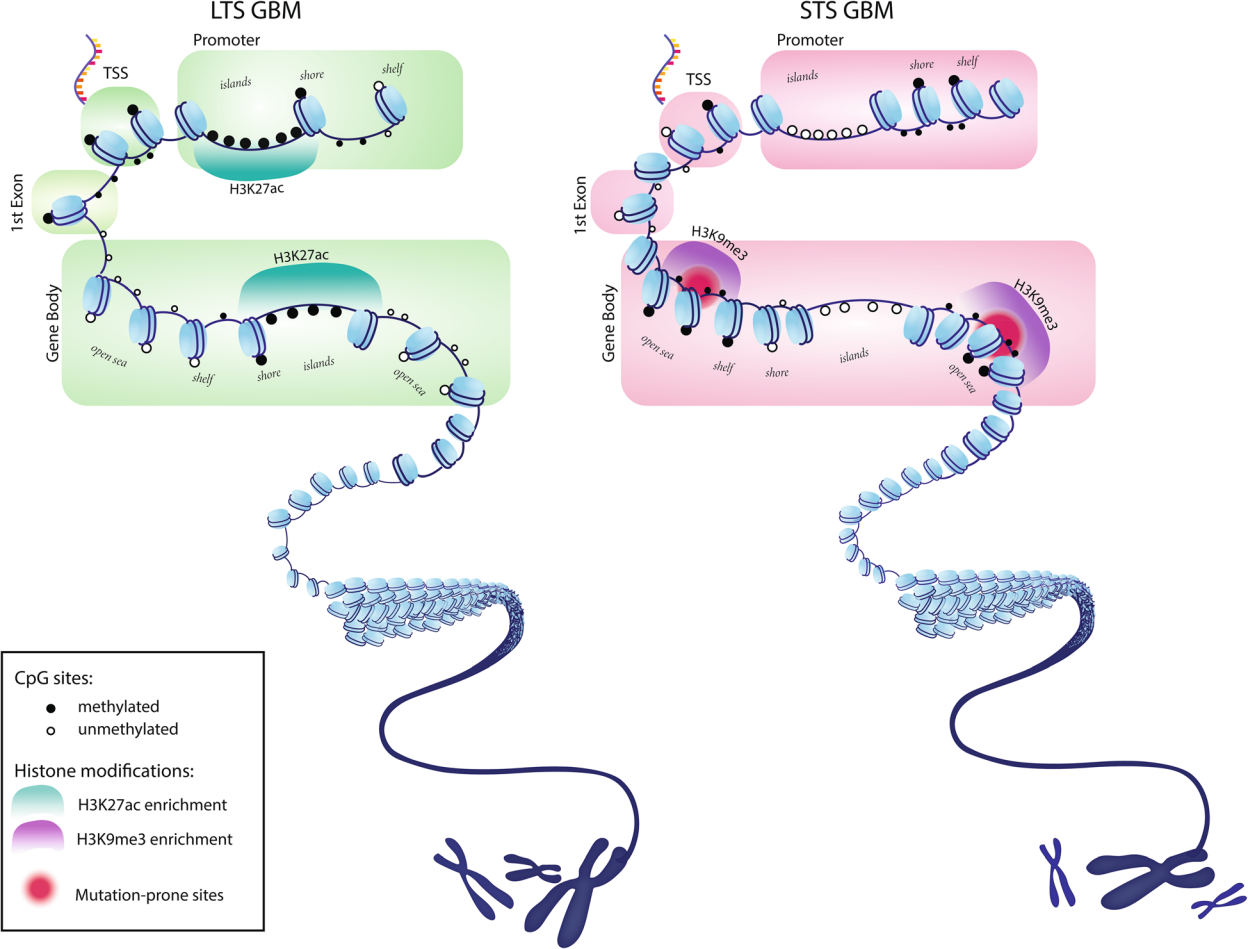
Genome-wide DNA methylation pattern of glioblastoma. The genomes of long-term survivors in glioblastoma are differentially methylated relative to short-term survival patients depending on CpG density: hypermethylation near CpG islands (CGIs) and hypomethylation far from CGIs (open sea). The hypermethylation at CGIs frequently occurs around regions with histone marks of active transcription such as H3K27ac, correlating with downregulation of gene expression in cancer progression pathways. The hypomethylated region at open sea are enriched with a histone mark of heterochromatin, H3K9me3. The rate of de novo mutation is high in this region when it is methylated, implying survival advantage of hypomethylation of the region in glioblastoma. In the figure, we highlighted genic regions such as first exon and gene body to emphasize potential effect of perturbed DNA methylation in glioblastoma

Our finding provides a clue on functional implications of global DNA methylation in survival outliers of glioblastoma, which are related to oncogenic pathways through the two distinct mechanisms of transcriptional suppression and somatic mutation depending on their genomic location (Fig. 5). The implication of DNA hypomethylation specific to long term survivors of glioblastoma call more attention to its dual aspects on both oncogenic contribution and survival benefits of patients.

## Additional files

Additional file 1: **Figures S1.** Classification of the sites in the 450 k bead array according to CGI. **Figure S2.** The cells and histone marks obtained from ENCODE. **Figure S3.** Volcano plot of differences between STS-GBM and LTS-GBM in DNA methylation level (beta). **Figure S4.** Proportion of the number of significant sites according to chromosomes. **Figure S5.** The relation between the ChIP-seq signal of H3K9me3 and the DNA methylation level (beta) for the hyper-methylated sites in U87 cell line. **Figure S6.** Enrichment of significant differential sites in gene regions. **Figure S7.** Distribution of Pearson correlation coefficients between DNA methylation level (beta) and gene expression measured by RNA-seq (FPKM) across GBM samples in TCGA. **Figure S8.** Distribution of Z-score of DNA methylation of the sample with somatic mutation. **Table S1.** Sample information. **Table S3.** Gene Ontology (GO) results of the hypo-methylated sites. (PDF 2060 kb)

Additional file 2 : **Table S2.** The list of 161,794 significant sites showing the DNA 145 methylationdifference between LTS and STS. (CSV 8954 kb)

## Data Availability

The datasets generated during the current study are available in the NCBI GEO (accession number: GSE114534).

## Abbreviations

CGI: CpG Island
GBM: Glioblastoma
G-CIMP: Glioma CpG Island Hypermethylator Phenotype
GO: Gene ontology
IDH: Isocitrate dehydrogenase
LTS-GBM: Long-term survivors of glioblastoma
MGMT: O6-methylguanine DNA methyltransferase
STS-GBM: Short-term survivors of glioblastoma
TSS: Transcription start sites

## References

[1] Amelot A, De Cremoux P, Quillien V, Polivka M, Adle-Biassette H, Lehmann-Che J, Francoise L, Carpentier AF, George B, Mandonnet E, Froelich S (2015). IDH-mutation is a weak predictor of long-term survival in glioblastoma patients. PLoS One.

[2] Burton EC, Lamborn KR, Feuerstein BG, Prados M, Scott J, Forsyth P, Passe S, Jenkins RB, Aldape KD (2002). Genetic aberrations defined by comparative genomic hybridization distinguish long-term from typical survivors of glioblastoma. Cancer Res.

[3] Burton EC, Lamborn KR, Forsyth P, Scott J, O’Campo J, Uyehara-Lock J, Prados M, Berger M, Passe S, Uhm J, O’Neill BP, Jenkins RB, Aldape KD (2002). Aberrant p53, mdm2, and proliferation differ in glioblastomas from long-term compared with typical survivors. Clin Cancer Res.

[4] Ceccarelli M, Barthel FP, Malta TM, Sabedot TS, Salama SR, Murray BA, Morozova O, Newton Y, Radenbaugh A, Pagnotta SM, Anjum S, Wang J, Manyam G, Zoppoli P, Ling S, Rao AA, Grifford M, Cherniack AD, Zhang H, Poisson L, Carlotti CG, Tirapelli DP, Rao A, Mikkelsen T, Lau CC, Yung WK, Rabadan R, Huse J, Brat DJ, Lehman NL, Barnholtz-Sloan JS, Zheng S, Hess K, Rao G, Meyerson M, Beroukhim R, Cooper L, Akbani R, Wrensch M, Haussler D, Aldape KD, Laird PW, Gutmann DH, Noushmehr H, Iavarone A, Verhaak RG (2016). Molecular profiling reveals biologically discrete subsets and pathways of progression in diffuse glioma. Cell.

[5] Charlet J, Duymich CE, Lay FD, Mundbjerg K, Dalsgaard Sorensen K, Liang G, Jones PA (2016). Bivalent regions of cytosine methylation and H3K27 acetylation suggest an active role for DNA methylation at enhancers. Mol Cell.

[6] Ehrlich M (2009). DNA hypomethylation in cancer cells. Epigenomics.

[7] ENCODE Project Consortium (2012). An integrated encyclopedia of DNA elements in the human genome. Nature.

[8] Geisenberger C, Mock A, Warta R, Rapp C, Schwager C, Korshunov A, Nied AK, Capper D, Brors B, Jungk C, Jones D, Collins VP, Ichimura K, Backlund LM, Schnabel E, Mittelbron M, Lahrmann B, Zheng S, Verhaak RG, Grabe N, Pfister SM, Hartmann C, von Deimling A, Debus J, Unterberg A, Abdollahi A, Herold-Mende C (2015). Molecular profiling of long-term survivors identifies a subgroup of glioblastoma characterized by chromosome 19/20 co-gain. Acta Neuropathol.

[9] Gerber NK, Goenka A, Turcan S, Reyngold M, Makarov V, Kannan K, Beal K, Omuro A, Yamada Y, Gutin P, Brennan CW, Huse JT, Chan TA (2014). Transcriptional diversity of long-term glioblastoma survivors. Neuro-oncology.

[10] Hartmann C, Hentschel B, Simon M, Westphal M, Schackert G, Tonn JC, Loeffler M, Reifenberger G, Pietsch T, von Deimling A, Weller M (2013). Long-term survival in primary glioblastoma with versus without isocitrate dehydrogenase mutations. Clin Cancer Res.

[11] Jones PA (2012). Functions of DNA methylation: islands, start sites, gene bodies and beyond. Nat Rev Genet.

[12] Krex D, Klink B, Hartmann C, von Deimling A, Pietsch T, Simon M, Sabel M, Steinbach JP, Heese O, Reifenberger G, Weller M, Schackert G (2007). Long-term survival with glioblastoma multiforme. Brain.

[13] Lin CY, Lovén J, Rahl PB, Paranal RM, Burge CB, Bradner JE, Lee TI, Young RA (2012). Transcriptional amplification in tumor cells with elevated c-Myc. Cell.

[14] Lu J, Cowperthwaite MC, Burnett MG, Shpak M (2016). Molecular predictors of long-term survival in glioblastoma Multiforme patients. PLoS One.

[15] Ma J, Hou X, Li M, Ren H, Fang S, Wang X, He C (2015). Genome-wide methylation profiling reveals new biomarkers for prognosis prediction of glioblastoma. J Cancer Res Ther.

[16] Mack SC, Hubert CG, Miller TE, Taylor MD, Rich JN (2016). An epigenetic gateway to brain tumor cell identity. Nat Neurosci.

[17] Makova KD, Hardison RC (2015). The effects of chromatin organization on variation in mutation rates in the genome. Nat Rev Genet.

[18] Maksimovic J, Gagnon-Bartsch JA, Speed TP, Oshlack A (2015). Removing unwanted variation in a differential methylation analysis of Illumina HumanMethylation450 array data. Nucleic Acids Res.

[19] Malzkorn B, Wolter M, Riemenschneider MJ, Reifenberger G (2011). Unraveling the glioma epigenome: from molecular mechanisms to novel biomarkers and therapeutic targets. Brain Pathol.

[20] Martinez R, Schackert G, Yaya-Tur R, Rojas-Marcos I, Herman JG, Esteller M (2007). Frequent hypermethylation of the DNA repair gene MGMT in long-term survivors of glioblastoma multiforme. J Neuro-Oncol.

[21] Millward CP, Brodbelt AR, Haylock B, Zakaria R, Baborie A, Crooks D, Husband D, Shenoy A, Wong H, Jenkinson MD (2016). The impact of MGMT methylation and IDH-1 mutation on long-term outcome for glioblastoma treated with chemoradiotherapy. Acta Neurochir.

[22] Mock A, Geisenberger C, Orlik C, Warta R, Schwager C, Jungk C, Dutruel C, Geiselhart L, Weichenhan D, Zucknick M, Nied AK, Friauf S, Exner J, Capper D, Hartmann C, Lahrmann B, Grabe N, Debus J, von Deimling A, Popanda O, Plass C, Unterberg A, Abdollahi A, Schmezer P, Herold-Mende C (2016). LOC283731 promoter hypermethylation prognosticates survival after radiochemotherapy in IDH1 wild-type glioblastoma patients. Int J Cancer.

[23] Morris TJ, Butcher LM, Feber A, Teschendorff AE, Chakravarthy AR, Wojdacz TK, Beck S (2014). ChAMP: 450k Chip analysis methylation pipeline. Bioinformatics.

[24] Nakagawa Yu, Sasaki Hikaru, Ohara Kentaro, Ezaki Taketo, Toda Masahiro, Ohira Takayuki, Kawase Takeshi, Yoshida Kazunari (2017). Clinical and Molecular Prognostic Factors for Long-Term Survival of Patients with Glioblastomas in Single-Institutional Consecutive Cohort. World Neurosurgery.

[25] Noushmehr H, Weisenberger DJ, Diefes K, Phillips HS, Pujara K, Berman BP, Pan F, Pelloski CE, Sulman EP, Bhat KP, Verhaak RG, Hoadley KA, Hayes DN, Perou CM, Schmidt HK, Ding L, Wilson RK, Van Den Berg D, Shen H, Bengtsson H, Neuvial P, Cope LM, Buckley J, Herman JG, Baylin SB, Laird PW, Aldape K (2010). Identification of a CpG island methylator phenotype that defines a distinct subgroup of glioma. Cancer Cell.

[26] Ostrom QT, Gittleman H, Xu J, Kromer C, Wolinsky Y, Kruchko C, Barnholtz-Sloan JS (2016). CBTRUS statistical report: primary brain and other central nervous system tumors diagnosed in the United States in 2009-2013. Neuro-Oncol.

[27] Parsons DW, Jones S, Zhang X, Lin JC-H, Leary RJ, Angenendt P, Mankoo P, Carter H, Siu IM, Gallia GL, Olivi A, McLendon R, Rasheed BA, Keir S, Nikolskaya T, Nikolsky Y, Busam DA, Tekleab H, Diaz LA, Hartigan J, Smith DR, Strausberg RL, Marie SKN, Shinjo SMO, Yan H, Riggins GJ, Bigner DD, Karchin R, Papadopoulos N, Parmigiani G, Vogelstein B, Velculescu VE, Kinzler KW (2008). An integrated genomic analysis of human glioblastoma multiforme. Science.

[28] Peng S, Dhruv H, Armstrong B, Salhia B, Legendre C, Kiefer J, Parks J, Virk S, Sloan AE, Ostrom QT, Barnholtz-Sloan JS, Tran NL, Berens ME (2017). Integrated genomic analysis of survival outliers in glioblastoma. Neuro Oncol.

[29] Prasanna Prateek, Patel Jay, Partovi Sasan, Madabhushi Anant, Tiwari Pallavi (2016). Radiomic features from the peritumoral brain parenchyma on treatment-naïve multi-parametric MR imaging predict long versus short-term survival in glioblastoma multiforme: Preliminary findings. European Radiology.

[30] Reifenberger G, Weber RG, Riehmer V, Kaulich K, Willscher E, Wirth H, Gietzelt J, Hentschel B, Westphal M, Simon M, Schackert G, Schramm J, Matschke J, Sabel MC, Gramatzki D, Felsberg J, Hartmann C, Steinbach JP, Schlegel U, Wick W, Radlwimmer B, Pietsch T, Tonn JC, von Deimling A, Binder H, Weller M, Loeffler M (2014). Molecular characterization of long-term survivors of glioblastoma using genome- and transcriptome-wide profiling. Int J Cancer.

[31] Rideout WM, Coetzee GA, Olumi AF, Jones PA (1990). 5-Methylcytosine as an endogenous mutagen in the human LDL receptor and p53 genes. Science.

[32] Rose NR, Klose RJ (2014). Understanding the relationship between DNA methylation and histone lysine methylation. Biochim Biophys Acta.

[33] Sarmiento JM, Mukherjee D, Black KL, Fan X, Hu JL, Nuno MA, Patil CG (2016). Do long-term survivor primary glioblastoma patients harbor IDH1 mutations?. J Neurol Surg A Cent Eur Neurosurg.

[34] Schuster-Bockler B, Lehner B (2012). Chromatin organization is a major influence on regional mutation rates in human cancer cells. Nature.

[35] Scott JN, Rewcastle NB, Brasher PM, Fulton D, MacKinnon JA, Hamilton M, Cairncross JG, Forsyth P (1999). Which glioblastoma multiforme patient will become a long-term survivor? A population-based study. Ann Neurol.

[36] Shinawi T, Hill VK, Krex D, Schackert G, Gentle D, Morris MR, Wei W, Cruickshank G, Maher ER, Latif F (2013). DNA methylation profiles of long- and short-term glioblastoma survivors. Epigenetics.

[37] Shinojima N, Kochi M, Hamada J, Nakamura H, Yano S, Makino K, Tsuiki H, Tada K, Kuratsu J, Ishimaru Y, Ushio Y (2004). The influence of sex and the presence of giant cells on postoperative long-term survival in adult patients with supratentorial glioblastoma multiforme. J Neurosurg.

[38] Sloan CA, Chan ET, Davidson JM, Malladi VS, Strattan JS, Hitz BC, Gabdank I, Narayanan AK, Ho M, Lee BT, Rowe LD, Dreszer TR, Roe G, Podduturi NR, Tanaka F, Hong EL, Cherry JM (2016). ENCODE data at the ENCODE portal. Nucleic Acids Res.

[39] Smrdel U, Popovic M, Zwitter M, Bostjancic E, Zupan A, Kovac V, Glavac D, Bokal D, Jerebic J (2016). Long-term survival in glioblastoma: methyl guanine methyl transferase (MGMT) promoter methylation as independent favourable prognostic factor. Radiol Oncol.

[40] Teschendorff AE, Marabita F, Lechner M, Bartlett T, Tegner J, Gomez-Cabrero D, Beck S (2013). A beta-mixture quantile normalization method for correcting probe design bias in Illumina Infinium 450 k DNA methylation data. Bioinformatics.

[41] Turcan S, Rohle D, Goenka A, Walsh LA, Fang F, Yilmaz E, Campos C, Fabius AWM, Lu C, Ward PS, Thompson CB, Kaufman A, Guryanova O, Levine R, Heguy A, Viale A, Morris LGT, Huse JT, Mellinghoff IK, Chan TA (2012). IDH1 mutation is sufficient to establish the glioma hypermethylator phenotype. Nature.

[42] Wang K, Li M, Hakonarson H (2010). ANNOVAR: functional annotation of genetic variants from high-throughput sequencing data. Nucleic Acids Res.

[43] Young MD, Wakefield MJ, Smyth GK, Oshlack A (2010). Gene ontology analysis for RNA-seq: accounting for selection bias. Genome Biol.

[44] Zhang W, Yan W, You G, Bao Z, Wang Y, Liu Y, You Y, Jiang T (2013). Genome-wide DNA methylation profiling identifies ALDH1A3 promoter methylation as a prognostic predictor in G-CIMP- primary glioblastoma. Cancer Lett.

