# Supplementary material for: Integrative analysis of DNA methylation suggests down-regulation of oncogenic pathways and reduced somatic mutation rates in survival outliers of glioblastoma
Source: Acta Neuropathol Commun. 2019 Jun 3;7:5. doi: 10.1186/s40478-019-0744-0 (PMC6545689; doi:10.1186/s40478-019-0744-0)
Supplement: Supplementary file 1 — Figures S1. Classification of the sites in the 450 k bead array according to CGI. Figure S2. The cells and histone marks obtained from ENCODE. Figure S3. Volcano plot of differences between STS-GBM and LTS-GBM in DNA methylation level (beta). Figure S4. Proportion of the number of significant sites according to chromosomes. Figure S5. The relation between the ChIP-seq signal of H3K9me3 and the DNA methylation level (beta) for the hyper-methylated sites in U87 cell line. Figure S6. Enrichment of significant differential sites in gene regions. Figure S7. Distribution of Pearson correlation coefficients between DNA methylation level (beta) and gene expression measured by RNA-seq (FPKM) across GBM samples in TCGA. Figure S8. Distribution of Z-score of DNA methylation of the sample with somatic mutation. Table S1. Sample information. Table S3. Gene Ontology (GO) results of the hypo-methylated sites. (PDF 2060 kb) [file 40478_2019_744_MOESM1_ESM.pdf]

Supplementary figure 1

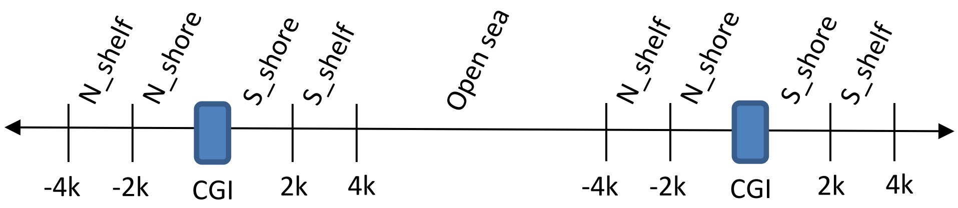

Classification of the sites in the 450k bead array according to CGI. The probes or sites were grouped into four categories according to the distance from the closest CGI: i) “island” if a probe is inside CGI, ii) “shore” if a probe is within 2000 bases, iii) “shelf” if a probe is between 2000 and 4000 bases, iv) “open sea” otherwise. Further categories were made by distinguishing 5’ and 3’ direction from the closest CGI denoted by ‘N_’ and ‘S_’ respectively such as ‘N_shore’ and ‘S_shore’.

Supplementary figure 2

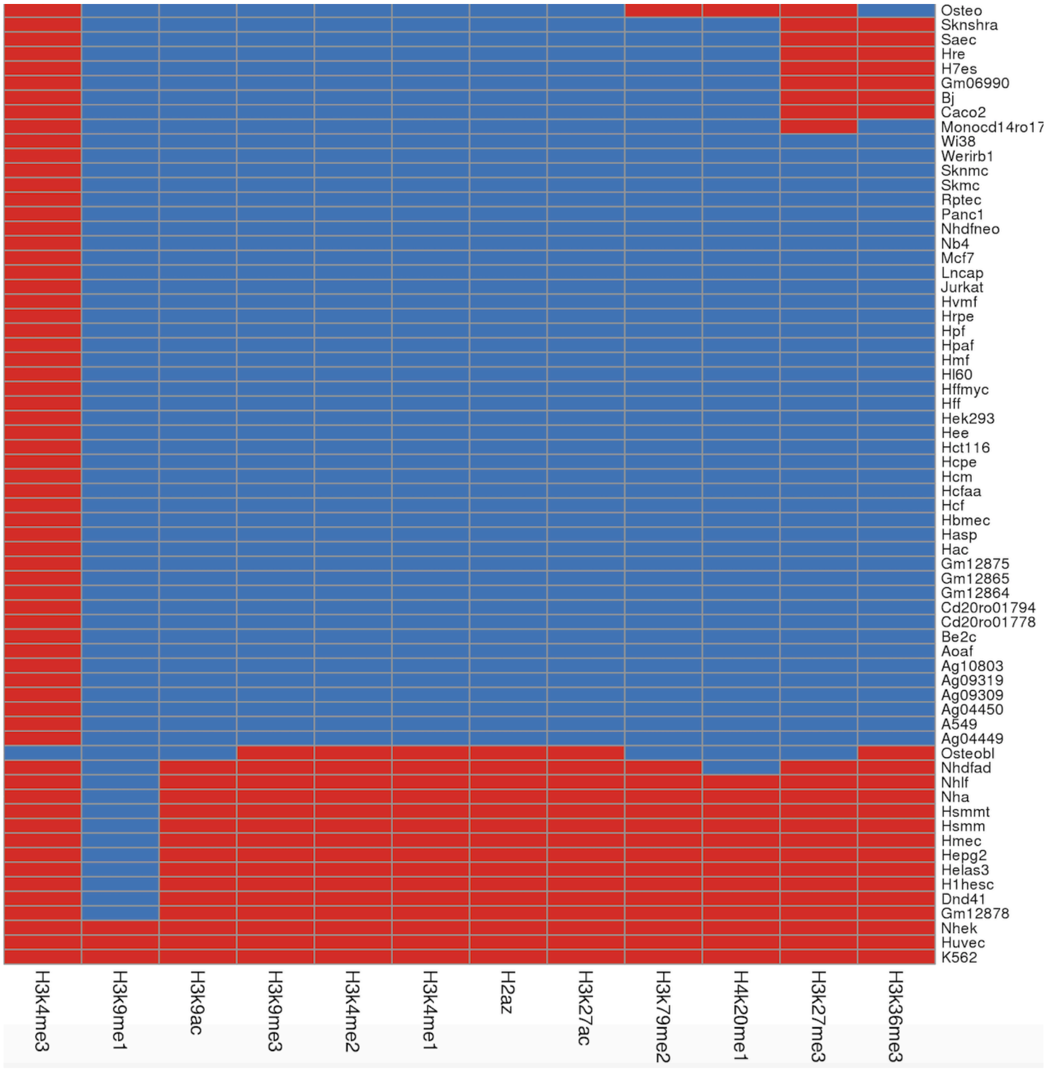

The cells and histone marks obtained from ENCODE. The red denotes the one used in the enrichment analyses while the blue indicates no data point available during this study.

Supplementary figure 3

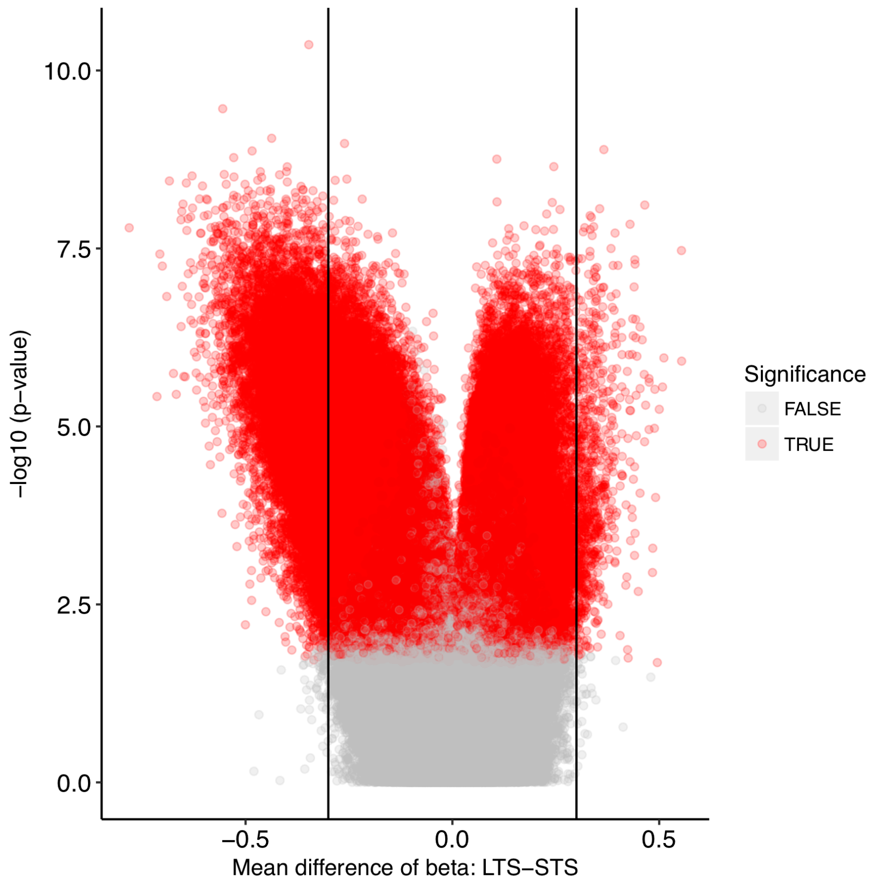

Volcano plot of differences between STS-GBM and LTS-GBM in DNA methylation level (beta). Each dot is a single CpG site measured by a probe. X axis is mean difference of beta values between STS-GBM and LTS-GBM groups. Y axis is statistical significance (p-value) in negative log (base: 10) scale. Red dots indicate significant sites while green dots correspond to sites without statistical significance, determined by the threshold of FDR of 0.01. Two vertical lines denote mean differences of -0.3 and 0.3 respectively.

Supplementary figure 4

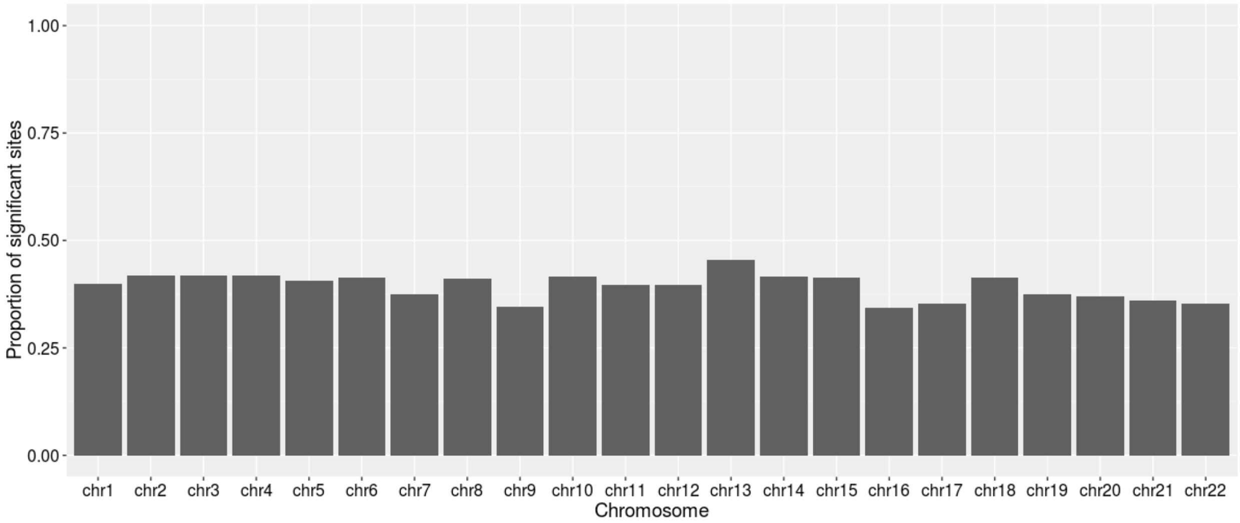

Proportion of the number of significant sites according to chromosomes. X axis is autosomal chromosome and Y axis is proportion of the significant sites that identified in discovery samples of LTS (n=17) and STS (n=12) in a given chromosome (number of significant sites divided by total number of sites that were tested).

Supplementary figure 5

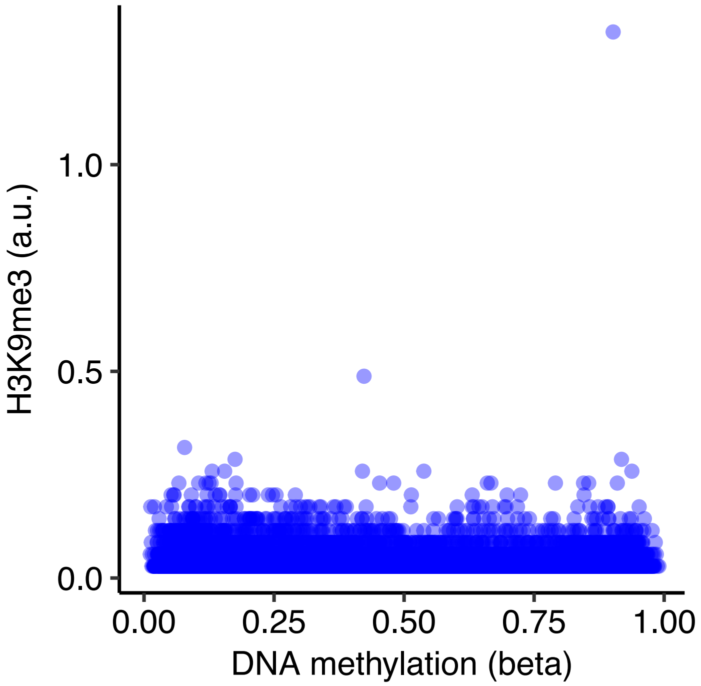

The relation between the ChIP-seq signal of H3K9me3 and the DNA methylation level (beta) for the hyper-methylated sites in U87 cell line. Each dot denotes the hypomethylated site.

Supplementary figure 6

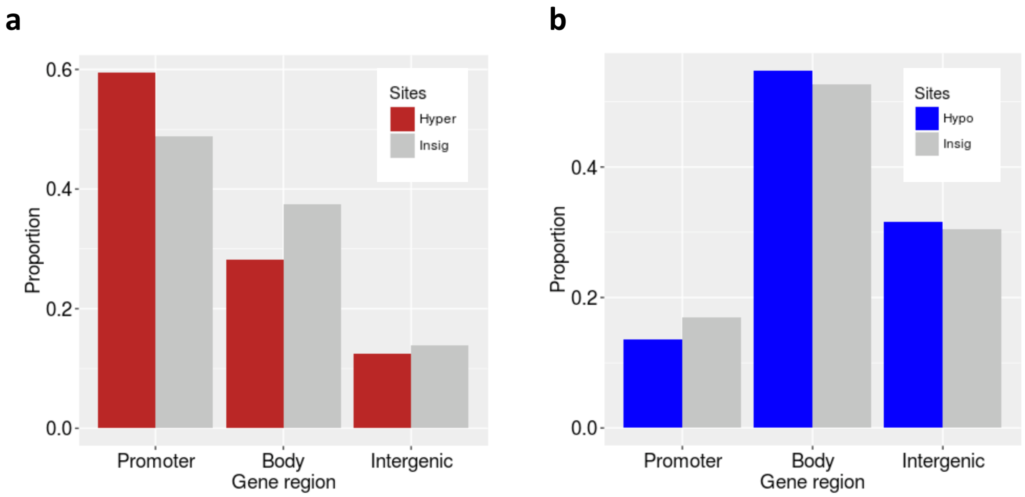

Enrichment of significant differential sites in gene regions. (a) Hypermethylated sites in island **(b)** Hypomethylated sites in open sea. Promoter is enriched with the hypermethylated site relative to gene body while gene body is enriched with the hypomethylated site relative to promoter (p-values<10^{-15} from Fisher exact tests in both cases).

Supplementary figure 7

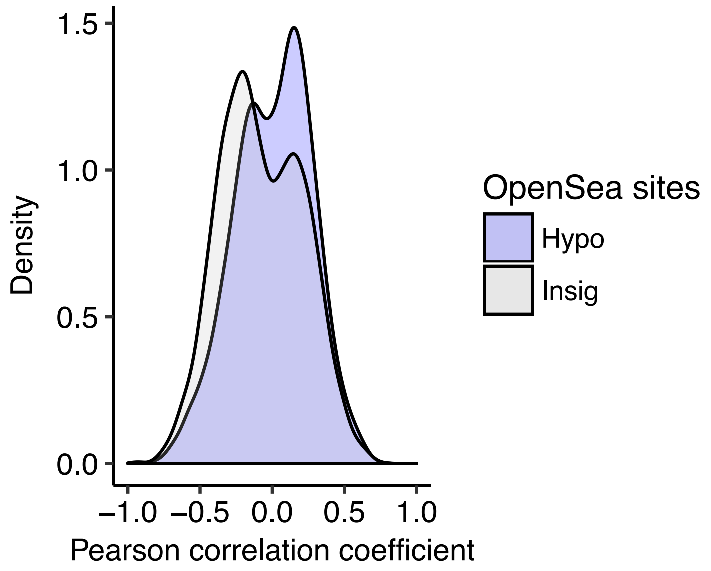

Distribution of Pearson correlation coefficients between DNA methylation level (beta) and gene expression measured by RNA-seq (FPKM) across GBM samples in TCGA. The blue describes the genes (# 3026) corresponding to the selected hypo-methylated sites while the gray shows the genes (# 4717) matching to the insignificant sites associated with the open sea.

Supplementary figure 8

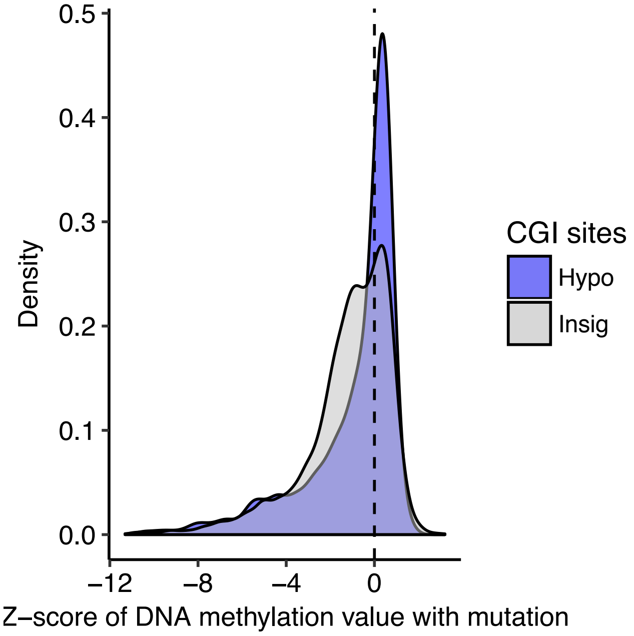

Distribution of Z-score of DNA methylation of the sample with somatic mutation. A Z-score, calculated by (beta-mean)/standard deviation represents the relative location of the sample with mutation in terms of DNA methylation level. The hypo-methylated showed the stronger shift to the right to the zero value of Z-score, meaning higher DNA methylation than the other samples on average, compared to the insignificant sites.

Supplementary table 1

Cohort		Case No.	Gender	Age	Survival	Management protocol	IDH1 IHC	MGMT MSP	PFS	Final status
Discovery cohort (SNU-LTS)	LTS (>3 years)	SNULTS-02	male	46 years	138 months	Resection-ACNU/CDDP-RT	negative	methylation	138 months	alive
		SNULTS-07	female	30 years	104 months	Resection-ACNU/CDDP-RT	negative	N.A.	104 months	alive
		SNULTS-10	female	48 years	95 months	Resection-ACNU/CDDP-RT	negative	N.A.	95 months	alive
		SNULTS-18	male	50 years	59 months	Resection-ACNU/CDDP-RT	negative	N.A.	N.A.	dead
		SNULTS-19	female	45 years	59 months	Resection-ACNU/CDDP-RT-TMZ	negative	methylation	20 months	dead
		SNULTS-20	female	38 years	58 months	Resection-RT-TMZ	negative	unmethylation	58 months	alive
		SNULTS-21	female	33 years	53 months	Resection-RT-TMZ	negative	N.A.	24 months	dead
		SNULTS-22	female	28 years	49 months	Resection-CCRT/TMZ-TMZ	negative	methylation	41 months	dead
		SNULTS-23	female	28 years	46 months	Resection-CCRT/TMZ	negative	unmethylation	46 months	alive
		SNULTS-25	female	57 years	44 months	Biopsy-CCRT/TMZ-TMZ	negative	unmethylation	N.A.	dead
		SNULTS-26	male	48 years	42 months	Resection-ACNU/CDDP-RT-TMZ	negative	unmethylation	N.A.	dead
		SNULTS-27	male	81 years	42 months	Resection-CCRT/TMZ-TMZ	negative	methylation	N.A.	dead
		SNULTS-29	male	41 years	41 months	Resection-CCRT/TMZ-TMZ	negative	methylation	N.A.	dead

		SNULTS-30	male	57 years	41 months	Resection-CCRT/TMZ-TMZ	negative	unmethylation	26 months	dead
		SNULTS-31	female	54 years	41 months	Resection-ACNU/CDDP-RT	negative	unmethylation	N.A.	dead
		SNULTS-33	female	57 years	38 months	Resection-RT-TMZ	negative	N.A.	N.A.	dead
		SNULTS-34	male	43 years	36 months	Resection-CCRT/TMZ-TMZ	negative	Unmethylated	27 months	dead
	STS (<1 year)	SNUSTS-01	male	55 years	40 weeks	Resection-CCRT/TMZ-TMZ	negative	methylation	26 weeks	dead
		SNUSTS-02	male	45 years	54 weeks	Resection-CCRT/TMZ-TMZ	negative	unmethylation	26 weeks	dead
		SNUSTS-03	female	64 years	52 weeks	Resection-CCRT/TMZ-TMZ	negative	methylation	N.A.	dead
		SNUSTS-04	female	68 years	26 weeks	Resection-CCRT/TMZ-TMZ	negative	unmethylation	N.A.	dead
		SNUSTS-05	male	70 years	52 weeks	Resection-CCRT/TMZ-TMZ	negative	unmethylation	N.A.	dead
		SNUSTS-06	male	42 years	37 weeks	Resection-CCRT/TMZ-TMZ	negative	methylation	31 weeks	dead
		SNUSTS-07	male	63 years	50 weeks	Resection-CCRT/TMZ-TMZ	negative	unmethylation	38 weeks	dead
		SNUSTS-08	male	62 years	37 weeks	Resection-CCRT/TMZ-TMZ	negative	unmethylation	20 weeks	dead
		SNUSTS-09	female	60 years	45 weeks	Resection-CCRT/TMZ-TMZ	negative	unmethylation	15 weeks	dead
		SNUSTS-10	male	59 years	52 weeks	Resection-CCRT/TMZ-TMZ	negative	methylation	35 weeks	dead
		SNUSTS-11	male	39 years	47 weeks	Resection-CCRT/TMZ-TMZ	negative	methylation	40 weeks	dead

		SNUSTS-12	male	61 years	23 weeks	Resection-CCRT/TMZ-TMZ	negative	unmethylation	N.A.	dead
Validation cohort (AUS)	LTS (>3 years)	AUS-01	male	56 years	55 months	Resection-CCRT/TMZ-TMZ/Bev	negative	unmethylation	38 months	dead
		AUS-02	male	62 years	86 months	Resection-CCRT/TMZ-TMZ	negative	methylation	N.A.	dead
		AUS-03	male	20 years	76 months	Resection-CCRT/Procarbaziine	negative	methylation	N.A.	dead
		AUS-04	female	36 years	66 months	Resection-TMZ/Procarbazine/Bev	negative	unmethylation	N.A.	dead
		AUS-05	male	61 years	83 months	Resection-CCRT/TMZ-TMZ/Procarbazine/Bev	negative	methylation	N.A.	dead
		AUS-07	male	34 years	135 months	Resection-CCRT/TMZ-TMZ	positive	unmethylation	N.A.	dead
		AUS-08	male	64 years	58 months	Resection-CCRT/TMZ-TMZ	N.A.	unmethylation	45 months	dead
		AUS-09	male	57 years	48 months	Resection-CCRT/TMZ-TMZ	N.A.	unmethylation	33 months	dead
		AUS-10	female	54 years	95 months	Resection-CCRT/TMZ-TMZ	N.A.	unmethylation	16 months	dead
		AUS-11	female	77 years	76 months	Resection-CCRT/TMZ-TMZ	N.A.	methylation	N.A.	dead
Validation cohort (TCGA patient ID)	LTS (>3 years)	TCGA-06-0125, TCGA-76-4932, TCGA-14-1450								
	STS (<1 year)	TCGA-14-1043, TCGA-14-0781, TCGA-32-1980, TCGA-14-1395, TCGA-06-6391, TCGA-81-5910, TCGA-19-5955, TCGA-12-5301, TCGA-76-4934, TCGA-76-6193, TCGA-06-5418, TCGA-76-4928, TCGA-12-5299, TCGA-76-6192, TCGA-74-6573, TCGA-06-5410, TCGA-76-4929, TCGA-06-5856, TCGA-06-5412, TCGA-76-4926, TCGA-19-1389, TCGA-76-4925, TCGA-28-5218, TCGA-06-6388, TCGA-06-6390, TCGA-28-6450, TCGA-19-5947, TCGA-06-0210,								

		TCGA-19-5951, TCGA-06-5411, TCGA-76-6285, TCGA-26-5135, TCGA-76-4931, TCGA-06-0190, TCGA-28-5215,<br>TCGA-28-5207, TCGA-06-5408, TCGA-06-0211, TCGA-14-0740

Sample information. Cohort: class of patient sample group (LTS; long-term survivor, STS; short-term survivor), Case No.: case-specific ID, Gender: male or female, Age: age at diagnosis in years, Diagnosis: histological diagnosis, Survival: survival period between first operation and death, IDH1 IHC: immunohistochemistry result of IDH1, MGMT MSP: MGMT methylation specific PCR, PFS: progression free survival, Final status: survival status at the last follow-up. For TCGA cohort, TCGA patient IDs are provided for reference. N.A. means “not available”.

Supplementary table 2 The list of 161,794 significant sites showing the DNA methylationdifference between LTS and STS. This is provided as a separate file (additional file 2) due to its size. The file incldues the following information on the sites: 450K bead array ID ("ProbeID"), Chromosome ("Chrom"), Coordinate ("Coord"), Gene name ("Gene"), CGI category of a site ("CGI_group"), TSS categroy of a site ("Gene_group"), Average DNA methylation level in discovery STS samples ("STS_Mean"), Average DNA methylation level in discovery LTS samples ("LTS_Mean"). The genomic coordinate is based on UCSC hg19.

Supplementary table 3

ID	Description	Number of genes (intersection/ total)	Significance (p-value, FDR)
GO:0002250	An immune response mediated by cells expressing specific receptors for antigen produced through a somatic diversification process, and allowing for an enhanced secondary response to subsequent exposures to the same antigen (immunological memory).	72 / 100	0, 0
GO:0050911	The series of events involved in the perception of smell in which an olfactory chemical stimulus is received and converted into a molecular signal.	135 / 302	0, 0
GO:0007031	A process that is carried out at the cellular level which results in the assembly, arrangement of constituent parts, or disassembly of a peroxisome. A peroxisome is a small, membrane-bounded	9 / 10	0.001, 0.051

	organelle that uses dioxygen (O2) to oxidize organic molecules.		

Gene Ontology (GO) results of the hypo-methylated sites. No: number, ID: GO ID, Description: GO Term description, Number of genes: “total” is the number of genes that are mapped in the 450K bead array in the GO term, “intersection” means the number of genes that have associations with the selected hyper-methylated sites, Significance: “p-value” was obtained from permutation test, “FDR” is False Discovery Rate.
